# Antifungal Therapy in Hematopoietic Stem Cell Transplant Recipients

**DOI:** 10.4084/MJHID.2016.039

**Published:** 2016-09-01

**Authors:** Alessandro Busca, Livio Pagano

**Affiliations:** 1 A.O.U. Città della Salute e della Scienza di Torino, Dipartimento di Oncologia, SSD Trapianto allogenico di cellule staminali, Torino, Italy; 2 Istituto di Ematologia, Università Cattolica del Sacro Cuore, Roma

## Abstract

Invasive fungal infections (IFI) represent a major hindrance to the success of hematopoietic stem cell transplantation (HSCT), contributing substantially to morbidity and infection-related mortality. During the most recent years several reports indicate an overall increase of IFI among hematologic patients, in particular, invasive aspergillosis, that may be explained, at least partially, by the fact that diagnoses only suspected in the past, are now more easily established due to the application of serum biomarkers and early use of CT scan. Along with new diagnostic options, comes the recent development of novel antifungal agents that expanded the spectrum of activity over traditional treatments contributing to the successful management of fungal diseases. When introduced in 1959, Amphotericin B deoxycholate (d-AmB) was a life-saving drug, and the clinical experience over 50 years has proven that this compound is effective although toxic. Given the superior safety profile, lipid formulations of AmB have now replaced d-AmB in many circumstances. Similarly, echinocandins have been investigated as initial therapy for IA in several clinical trials including HSCT recipients, although the results were moderately disappointing leading to a lower grade of recommendation in the majority of published guidelines. Azoles represent the backbone of therapy for treating immunocompromised patients with IFI, including voriconazole and the newcomer isavuconazole; in addition, large studies support the use of mold-active azoles, namely voriconazole and posaconazole, as antifungal prophylaxis in HSCT recipients.

The aim of the present review is to summarize the clinical application of antifungal agents most commonly employed in the treatment of IFI.

## Introduction

Bone marrow, peripheral blood stem cells, and umbilical cord blood transplantation are medical procedures that are widely used to treat diseases once thought incurable. Since the first human bone marrow transplant in the 1950s, over 1 million procedures have been completed worldwide, and the number of transplants performed each year is now close to 70.000. Hematopoietic stem cell transplantation (HSCT) has been used to treat a wide variety of malignant and non-malignant hematological disorders including leukemia, lymphomas, and aplastic anemia, and indications are expanding.

HSCT is a procedure that restores stem cells that have been destroyed by a preparative regimen including chemotherapy with or without total-body irradiation usually delivered before stem cell infusion to optimize tumor cell kill and, in the case of allogeneic HSCT, immunosuppress the recipient to prevent graft rejection. In addition, allogeneic HSCT recipients may receive immunosuppressive agents, namely calcineurin inhibitors, for a prolonged period after transplant to mitigate the graft-versus-host reaction. According to these considerations, HSCT is associated with a profound immune deficiency resulting in an increased propensity to develop opportunistic infections, in particular, invasive fungal infections (IFI).

Indeed, the last two decades have witnessed an increasing incidence of life-threatening systemic fungal infections in immunocompromised patients, and the epidemiology of IFI in HSCT recipients is undergoing significant changes. Table 1 summarizes the studies published over the last ten years on the epidemiology of IFI in patients receiving HSCT.

The perception of an increase in mold infections has been confirmed by several studies recently published.^1^ The epidemiology of invasive aspergillosis (IA) has changed owing to the use of alternative sources of harvested stem cells, new regimens employed to decrease rejection and graft versus host disease (GVHD) and aggressive therapeutic modalities.^2^–^4^ In patients with autologous HSCT, the frequency of invasive aspergillosis (IA) has decreased due to more rapid engraftment,^5^ while the use of peripheral stem cells in allogeneic HSCT may be associated with beneficial engraftment at the theoretical cost of an increased incidence of GVHD.^6^ The recipients of cord blood and grafts selected for CD34+ cells have a higher risk for IA early after transplantation.^3^ These observations have been confirmed by two recent studies. Girmenia et al.^7^ investigated the epidemiology of IFI in a cohort of 1858 allogeneic HSCT recipients showing that grafts from an unrelated donor or umbilical cord blood were associated in multivariate analysis with a high risk of early IFI occurring before day 40. Similarly, Sun et al.^8^ demonstrated that the cumulative incidence of IFI in autologous HSCT patients, recipients of HLA-matched related, haploidentical, and unrelated HSCT was 3.5%, 4.3%, 13.2% and 12.8% respectively.

Given the high mortality rate reported in this patient population, the early diagnosis of IA remains a clinical challenge: the standard is limited to the correlation of the signs and symptoms of the disease with histopathologic detection of the organism. However, clinical circumstances make this strategy unattainable for many patients. The availability of the newer non-culture-based methods, including noninvasive serologic techniques (galactomannan and β-D-glucan assays) and molecular diagnostics, have become part of the diagnostic strategy in which they are combined with other tools such as the HR-CT scan.^9^

Along with new diagnostic options, comes the recent development of novel antifungal agents that expanded the spectrum of activity over traditional treatments contributing to the successful management of fungal diseases.

## Antifungal Treatment

The changing epidemiology of IA in combination to the recent advances in antifungal agents and diagnostic tools facilitating the early recognition of IFI led to redefining the approach to prevention and early treatment of IA in immunocompromised patients. At the present, four strategies may be identified as follows:

1. Prophylaxis may be considered as the first step and consists of the administration of antifungal agents at the onset of a period of high risk of infection, traditionally the beginning of neutropenia or the start of conditioning regimen in HSCT recipients. Antifungal prophylaxis should be considered as a therapeutic option designed to reduce the mortality and morbidity associated with invasive fungal infection, however, even among highly immunocompromised patients, most will not develop an IA.
2. Empirical treatment includes the initiation of an antifungal regimen in patients with signs or symptoms suggestive, even not fully documenting IA, and this is typically the case of neutropenic patients with persistent fever (generally 4–7 days in duration) despite the administration of broad-spectrum antibiotics. However, it has become quite clear that fever is a less than an adequate surrogate for evaluating those patients who are in need of antifungal therapy and have IA.
3. Diagnostic driven therapy aims to treat IA by radiologic studies and laboratory markers that might be helpful to recognize patients with fungal infection at an early phase of the disease.
4. Treatment of established infection applies to those patients who have the diagnosis of a proven fungal infection based on the EORTC/MSG criteria.

## Antifungal Prophylaxis

### Meta-analysis studies

Ziakas et al...^10^ evaluated 20 studies including 4823 patients receiving HSCT. Overall, the risk for IFI while on prophylaxis was 5.1%. The risk of IFI, systemic candidiasis and the need for empiric antifungal treatment was significantly reduced in patients receiving fluconazole compared with patients receiving placebo. Itraconazole was more effective than fluconazole for the prevention of aspergillosis at the expense of more frequent withdrawals. Micafungin was marginally more effective than fluconazole for the prevention of mold infections and IA and reducing the need for empiric antifungal therapy. Voriconazole showed marginally significant effects compared with fluconazole regarding IA and the need for empiric treatment. Voriconazole compared with itraconazole and posaconazole compared with amphotericinB were better regarding empirical antifungal treatment.

Xu et al.^11^ analyzed 17 studies including 5122 patients. The new mold-active agents, namely posaconazole, voriconazole, and micafungin, have reduced the incidence of IFI compared to fluconazole and itraconazole; in addition, posaconazole and voriconazole have reduced transplant-related mortality significantly.

Similarly, Bow et al.^12^ showed that posaconazole, voriconazole reduced the risk of proven/probable IFI compared to fluconazole and itraconazole.

### Clinical Trials

Table 2 describes the main clinical studies on antifungal prophylaxis in HSCT recipients.

#### Fluconazole

The first prospective randomized study evaluating the efficacy of fluconazole vs. placebo in patients receiving autologous/allogeneic HSCT was published in 1992.^13^ The results of this study showed that 2.8% of patients receiving fluconazole developed IFI, compares to 15.8% of those receiving placebo. The reduced rate of infection for the fluconazole group resulted in fewer IFI-related deaths (1/179 vs. 10/177; p<0.001), although there was no difference in the overall survival.

Another prospective randomized, double-blind study examined the efficacy of fluconazole 400 mg given prophylactically for 75 days post-transplant (autologous and allogeneic HSCT) compared to placebo.^14^ The results of this study showed that the rate of systemic fungal infections was significantly lower in the fluconazole arm as compared to placebo-treated patients (7% vs. 18%, p 0.004).^14^,^15^ The results of this study demonstrated an overall mortality benefit, as 17.5% more patients in the fluconazole arm survived until eight years after related and unrelated HSCT, and was the first to show that the prophylaxis with fluconazole is capable in patients allografted not only to prevent infections but also to affect the survival.

#### Itraconazole

An open-label, multicenter randomized trial comparing the efficacy and safety of itraconazole with fluconazole in 140 patients receiving an allogeneic HSCT was published in 2003.^16^ The results of this study showed that itraconazole resulted in fewer proven IFI (9% vs. 25%, p=0.01) and fewer deaths were related to fungal infection in patients given itraconazole (9%) than in patients given fluconazole (18%, p=0.13). More frequent gastrointestinal side effects were observed in patients given itraconazole as compared to fluconazole (24% vs. 9%).

The Seattle group reported the results of a large trial in 304 patients receiving an allogeneic HSCT, who were randomized to receive fluconazole (400 mg/day) or itraconazole (oral solution or iv) for 180 days after HSCT.^17^ The cumulative incidence of proven or probable IFI was not different between the two arms (fluconazole 16% vs. itraconazole 13%, p=0.46). Itraconazole provided better protection against invasive mold infections (fluconazole 12% vs. itraconazole 5%, p=0.03), but similar protection against candidiasis (3% vs. 2%, p=0.69), however, no survival benefit was seen with itraconazole. More patients in the itraconazole arm developed hepatoxicities, and more patients discontinued the drug because of toxicities or gastrointestinal intolerance (36% vs. 16%, p< 0.001).

#### Voriconazole

Wingard et al.^18^ published the results of a prospective randomized trial comparing voriconazole (200 mg BID iv/po for 100 days or 180 days in case of steroid treatment) vs. fluconazole (400 mg QD) in allogeneic HSCT recipients. The incidence of proven-probable or presumptive IFI was not different between the two arms, while the incidence of aspergillosis was marginally reduced in patients who received voriconazole. A subgroup analysis of patients with AML showed a significantly reduced incidence of IFI (8% vs. 21%, p 0.04) and a better fungal-free survival (78% vs. 61%, p 0.04) in patients receiving voriconazole.

Similar results have been reported in a second study^19^ comparing itraconazole and voriconazole. The incidence of IFI and overall survival were superimposable in the two treatment arms (voriconazole 1.3% vs. itraconazole 2%), while patients in the itraconazole group were able to receive the drug at least 30 days less than patients in the voriconazole group.

#### Posaconazole

This agent has been compared with oral fluconazole for prophylaxis of IFI in 600 patients with grade II–IV acute GVHD or extensive chronic GVHD (20). The incidence of IFI was not significantly different in the two study groups (5.3% posaconazole vs. 9% fluconazole, p=0.07), while posaconazole prophylaxis resulted in a lower number of invasive aspergillosis (2.3% posaconazole vs. 7% fluconazole, p=0.006). The mean time to onset of invasive fungal infection was 102 days in the posaconazole arm and 88 days in the fluconazole arm. The number of deaths due to proven or probable IFI was lower in the posaconazole group than in the fluconazole group (1% vs. 4%, p=0.46), although the overall mortality was similar in the two groups.

Several real life (retrospective) studies have been subsequently published, confirming the efficacy of prophylaxis with posaconazole in the setting of allogeneic HSCT.^21^–^23^

#### Echinocandins

A large number of studies have evaluated the efficacy of different echinocandins to prevent IFI in patients receiving HSCT.

The efficacy and safety of micafungin have been investigated in a large randomized, double-blind, comparative phase III trial for the prophylaxis of IFI in HSCT patients in comparison with fluconazole.^24^ Overall, the efficacy defined as the absence of proven-probable-suspected IFI was greater with micafungin than with fluconazole (80% vs. 73%, p 0.03). There was a nonsignificant trend toward a reduced incidence of invasive aspergillosis, although the absolute number of events was remarkably low in both arms (n=1 micafungin; n=7 fluconazole), possibly due to the inclusion of a large proportion of low-risk patients (70% autologous HSCT). Empirical antifungal therapy was required in fewer patients treated with micafungin than with fluconazole (15.1% versus 21.4%, respectively; *p* = 0.024).

Subsequent studies have confirmed the efficacy of micafungin in comparison to standard azoles (Table 2). One issue arising from published studies is the optimal dose of micafungin for prophylaxis of IFI in HSCT recipients. In fact, different dosages have been used in clinical trials, spanning from 50 mg up to 150 mg per day. Lagebrake et al.^25^ have analyzed the dose of 50 mg, 100 mg, 150 mg of micafungin as antifungal prophylaxis: the rate of IFI did not result different according to the doses, nor was different the incidence of side effect; a nonsignificant trend toward a greater need for empirical treatment has been observed with the lowest dose of 50 mg.

#### Polyenes

The role of polyenes as antifungal prophylaxis in HSCT recipients has been investigated in few studies (Table 2).

El-Cheikh et al.^26^ reported the results of e retrospective study in which liposomal-Amphotericin B (L-AmB), administered at the dose of 7.5 mg/Kg once a week in patients with acute or chronic GVHD, was compared to a historical control group of patients who received different prophylactic regimens (fluconazole in 71% of the cases). The incidence of IFI was reduced (8% vs. 36%, p 0.008) as well as the fungal related mortality (0% vs. 14%, p 0.005) in patients who received L-AmB, while overall survival was not statistically different. Otherwise, Luu Tran^27^ et al., did not find any significant benefit with the use of L-AmB (3 mg/Kg one a week) when compared to echinocandins and triazoles.

According to the published studies, several guidelines have provided recommendations on antifungal prophylaxis in patients candidates to HSCT (Table 3).

## Empirical Treatment

The rationale for empiric antifungal therapy is based on autopsy studies showing the role of IFI as the cause of death in neutropenic patients^28^ and on clinical observations defining the importance of early treatment in the prognosis of IFI.^29^ These findings were supported by several studies showing a decreased mortality due to IFI in patients receiving empiric AmB as compared with historical controls where antifungal therapy was initiated upon documentation.^30^–^33^

The first evidence that empiric antifungal treatment in neutropenic patients with a persistent fever might have beneficial effects has been shown in two prospective studies published in early’ 80^34^ comparing deoxycholate-AmB (d-AmB) versus no therapy. Nevertheless, these studies, when re-evaluated on the basis of the current knowledge, may be largely criticized and do not have the statistical power necessary to detect differences between the groups analyzed.

A consistent number of studies have analyzed the efficacy of different compounds used as an empirical treatment in hematologic patients with neutropenic fever, but none of these studies included HSCT recipients only (Table 4).

### Azoles

Three studies have compared fluconazole to AmB for empiric antifungal therapy in immunocompromised patients. Two of them included few patients,^35^,^36^ while the third included 317 neutropenic patients with persistent or recrudescent fever despite 4 or more days of antibacterial therapy who were randomly assigned to receive either fluconazole (400 mg intravenously once daily) or d-AmB (0.5 mg/kg once daily).^37^ A satisfactory response (at the end of therapy if the patient was afebrile, had no clinical or microbiological evidence of fungal infection, and did not require study termination due to lack of efficacy, drug toxicity, or death) occurred in 68% of the patients treated with fluconazole and in 67% of patients treated with d-AmB. Progressive or new fungal infections during therapy occurred in 13 (8%) patients treated with fluconazole and in 10 (6%) patients treated with d-AmB. Adverse events related to study drug occurred more often in patients treated with d-AmB (81%) than patients treated with fluconazole (13%, *P* = 0.001). Overall mortality (17% of patients treated with fluconazole versus 21% of patients treated with d-AmB) and mortality from fungal infection (4% of patients treated with fluconazole versus 3% of patients treated with d-AmB) were similar in each study group.

Itraconazole has been compared to conventional AmB in 384 neutropenic patients with cancer who had a persistent fever.^38^ The overall response rate was 47% in the itraconazole group and 38% in the AmB group, but fewer drug-related adverse events occurred in the itraconazole group than the AmB group (5% vs. 54%, p=0.001). Breakthrough fungal infections occurred in 5 patients in each group. In conclusion, the results of this study demonstrated that itraconazole and AmB have equivalent efficacy as empirical antifungal therapy. However, itraconazole is associated with significantly less toxicity.

Voriconazole has been compared to L-AmB in a prospective randomized multi-institutional trial.^39^ Analysis of the 5 composite end points (no breakthrough fungal infections; survival 7 days after end of therapy; no discontinuation of therapy prematurely; resolution of fever during neutropenia; complete or partial response of patients with baseline fungal infections) favoured L-AmB for all variables except the prevention of breakthrough fungal infections (1.9% in the voriconazole group vs 5% in the L-AmB group, p=0.02). A subgroup analysis of patients receiving an allogeneic HSCT (18% of the patients in both study groups) showed that breakthrough fungal infections occurred in 1.4% of the patients treated with voriconazole and 9.2% of the patients treated with L-AmB. Overall success rates were 23.7% in the voriconazole group and 30% in the L-AmB group, however, voriconazole did not fulfill the protocol-defined criteria for noninferiority to LAmB with respect to overall response to empirical therapy.

### AmB lipid formulations

In 1999, Walsh et al.^40^ first challenged the assumption that conventional AmB is the optimal antifungal treatment for patients with persistent fever, in a large randomized trial including 702 patients with persistent fever and neutropenia. L-AmB was as effective as d-AmB when success was analyzed on the basis of the composite five end points: the rates of successful treatment were similar (L-AmB 50% vs. d-AmB 49%) as well as survival (L-AmB 93% vs. d-AmB 90%), resolution of fever (58% in both groups) and discontinuation of the study drug (L-AmB 14% vs. d-AmB 19%); by contrast, there were fewer breakthrough fungal infections among patients receiving L-AmB (3%) than among those who received d-AmB (8%). With L-AmB fewer patients had infusion-related toxicity and nephrotoxicity. It should be underscored that several aspects of this study have been criticized, including the fact that d-AmB was administered at a low dose (0.6 mg/Kg/d) and the absence of salt loading for prevention of d-AmB nephrotoxicity.

In 1997 Prentice^41^ and coworkers published the results of two prospective open-label randomized trials comparing d-AmB at the dose of 1 mg/Kg/d (102 patients) with L-AmB 1 mg/Kg/d (L-AmB 1:118 patients) and L-AmB 3 mg/Kg/d (L-AmB 3:118 patients) for empirical treatment of fever in neutropenic adults and children. Efficacy was defined as defervescence without the development of new fungal infections. Patients who received LAmB had a 2–6-fold decrease in the incidence of drug-related side effects (p< 0.01). More importantly, treatment success was observed in 49% of patients in the d-AmB arm, 58% and 64% of L-AmB 1 and L-AmB 3 arms respectively (p=0.09); L-AmB 3 was significantly more efficacious than d-AmB (p=0.03).

### Echinocandins

Caspofungin has been tested for empirical antifungal therapy in the setting of a double-blind, non-inferiority study design where the study drug has been compared to L-AmB in 1123 patients.^42^ Caspofungin was found to be non-inferior to L-AmB (overall success rate 33.9% for caspofungin vs. 33.7% for L-AmB), with an advantage among patients with baseline documented infections (successful treatment 52% vs. 26% respectively); the proportion of patients who survived 7 days after therapy was greater in the caspofungin group (93% vs. 89% respectively) and premature study discontinuation for toxicity or lack of efficacy occurred less often in the caspofungin group than in the L-AmB group (10% vs 14% respectively). In a subgroup analysis of patients who received an allogeneic HSCT, a favorable response was observed in 43% of the patients treated with caspofungin and 37% of the patients treated with L-AmB. The excellent toxicity profile of caspofungin was demonstrated in this study: fewer patients had a nephrotoxic effect (3% vs. 11% respectively) and infusion-related events (35% vs. 52% respectively).

## Diagnostic-Driven Therapy (DDT)

The development of non-cultured based microbiological and radiological diagnostic tests that are rapid, sensitive and specific made possible an earlier diagnosis of IA. The incorporation of these tests in the routine management of neutropenic patients has the potential for targeting patients in true need of antifungal therapy. According to these statements, DDT aims to treat a suspected early IFI on the basis of radiologic studies, laboratory markers or both rather than fever alone.

The potential impact of this therapeutic approach has been explored in several studies, where antifungal treatment has been guided by the galactomannan test (GM),^43^ PCR,^44^ CT imaging,^45^ clinical criteria^46^ or a combination of clinical work-up and diagnostic tests.^47^,^48^

However, only a minority of these studies refer to patients receiving HSCT. Dignan FL et al.^45^ used an early treatment strategy based on CT scan in 99 allogeneic HSCT recipients. Interestingly, 17% of the patients received antifungal therapy based on radiologic imaging compared to 54% of the patients who would have received antifungal treatment if an empirical approach was used. Similar results have been reported by Oshima K et al.^49^ with the use of a treatment strategy in which antifungal agents were initiated when patients had a positive serum test and/or radiologic imaging suggestive of IFI. Hebart et al.^44^ performed a randomized trial comparing PCR-based treatment and empirical antifungal therapy with L-AmB in 408 patients undergoing allogeneic HSCT. Patients randomized to PCR-based strategy had PCR screening planned twice weekly while inpatients and once weekly after discharge until day +100. L-AmB was initiated in those cases with two consecutive positive PCR results and in the empirical treatment group after five days of febrile neutropenia not responding to broad-spectrum antibiotics. Eleven patients in the PCR-based strategy were diagnosed with IFI compared with 16 in the empirical treatment group. A reduction in early mortality was documented until day +30 for patients receiving PCR-based antifungal therapy (4 deaths vs. 13 deaths in the empirical treatment group, p=0.03).

## Treatment of established invasive aspergillosis

Several studies evaluated the efficacy of different antifungal agents in patients with hematologic malignancies, but the number of HSCT recipients included in these studies, ranges between 20–30%, except one study analyzing the efficacy of caspofungin (Table 5).

Herbrecht et al.^50^ evaluated Caspofungin as first-line therapy of proven-probable IA in 24 allogeneic HSCT recipients. Among the 24 eligible patients, a favorable response was reported in 42% of the cases, and overall survival at 12 weeks was 50%. Although these results may seem disappointing, responses compare favorably to those reported with the use of voriconazole (overall response 32%) and L-AmB (overall response 47%) in the subgroup of patients receiving HSCT.

More recently, Marr et al.^51^ evaluated the safety and efficacy of voriconazole combined with Anidulafungin compared with voriconazole monotherapy for the treatment of IA. Mortality rates at six weeks were 19.3% for combination therapy and 27.5% for monotherapy (p 0.087). In a post hoc analysis of patients with a diagnosis based on radiological abnormalities and GM positivity, the 6-week mortality was significantly lower in combination therapy compared to monotherapy 15.7% vs. 27.3%, p 0.037). The safety profile was similar in the two treatment groups.

Isavuconazole is a new extended-spectrum prodrug triazole with efficacy for IA and mucormycosis. The SECURE study^52^ was a randomized, double-blind trial which evaluated the noninferiority of isavuconazole compared with voriconazole for the primary treatment of IFI. The majority of patients had an underlying hematologic malignancy (82% isavuconazole; 86% voriconazole). The primary end point of crude all-cause-mortality at six weeks was 18.6% in the isavuconazole arm and 20.2% in the voriconazole arm, demonstrating noninferiority of isavuconazole to voriconazole. Significantly fewer patients reported events considered drug-related by the investigator for isavuconazole than for voriconazole (42% *vs.* 60%; p<0.001).

Based on the published studies, the ECIL-6 guidelines assigned the higher strength of recommendation to voriconazole, isavuconazole (both AI) and L-AmB (BI); ABLC received BII, caspofungin CII and the combination voriconazole-anidulafungin CI.

## Final Remarks

Hematologic patients receiving allogeneic HSCT should be considered at risk for opportunistic infections including fungal infections, due to the presence of a severe impairment of immune responses that might persist even for several months after transplantation. In this respect, antifungal prophylaxis may be considered as the first step for a correct approach to patients undergoing HSCT. Epidemiologic modifications of fungal infections occurring in HSCT recipients advise the use of mold-active agents in high-risk patients, namely HSCT from alternative donors and patients with GVHD requiring high dose steroids. The advent of new diagnostic tools allows a timely and precise diagnosis of IFI. Accordingly, the therapeutic approach should always more often triggered by a positive diagnostic investigation, although an early empiric treatment seems to be justified awaiting the results of the diagnostic workup. Azoles resistance is an emerging issue requiring particular attention by clinicians. In this respect, the therapeutic armamentarium may be considered sufficiently large, since we have molecules such as L-AmB with great efficacy and no evidence of an increase of resistance, new compounds such as isavuconazole with a wide spectrum of activity, and new combinations such as voriconazole and anidulafungin that might be reserved for salvage treatments.

## Figures and Tables

**Table 1:** Epidemiology of invasive fungal infections (IFI) in patients receiving hematopoietic stem cell transplantation (HSCT).

Reference	Type of study Timeframe	No. Patients	HSCT	Prophylaxis	IFI rate	mortality
Garcia-Vidal C. CID 2008 (53)	Retrospective 1998–2002	1248	Allo	-	IMI 13.1%	-
Pagano L. SEIFEM CID 2007 (54)	Retrospective 1999–2003	3228	Auto 60% Allo 40%	Fluconazole 39% Itraconazole 21%	Auto 1.2% Allo 7.8%	AM Auto 14% AM Allo 77%
Mikulska M. BMT 2009 (55)	Retrospective 1999–2006	306	Allo	Fluconazole	IA 15%	AM IA 67%
Neofytos D. PATH Alliance CID 2009 (56)	Prospective 2004–2007	234	Auto 31% Allo 69%	-	-	IA 21.5%
Kontoyiannis DP. TRANSNET CID 2010 (57)	Prospective 2001–2006	875	Auto Allo	-	Auto 1.2% Allo-MSD 5.8% Allo-MUD 7.7%	-
Nucci M. CMI 2013 (58)	Prospective 2007–2009	700	Allo 54% Auto 46%	Fluconazole Allo 81%-Auto 73%	Allo IFI 11.3%-IA 2.3% Auto IFI 1.9%-IA 0%	-
Omer AK. BBMT 2010 (59)	Retrospective 2000–2010	271	Allo	Fluconazole 90%	IFI 15%	AM 33%
Atalla A TID 2015 (60)	Prospective 2007–2009	345	Allo	Fluconazole 89%	IMI 8.1%	-
Girmenia C. GITMO BBMT 2014 (7)	Prospective 2008–2010	1858	Allo	Fluconazole 75% Mold-active 14%	IFI 8.8%	AM 19%
Sun Y BBMT 2015 (8)	Prospective 2011	1401	Allo 75% Auto 25%	Fluconazole 61% Itraconazole 22% Voriconazole 19%	Allo 8.9% Auto 4%	Proven IFI 31% Probable IFI 22%
Corzo-Leon DE. Mycoses 2015 (61)	Retrospective 2002–2011	378	Allo	Fluconazole voriconazole	IA 7.9%	IA 52%
Liu Y-C. J Mic Imm Infec 2015 (62)	Retrospective 2002–2013	421	Allo	Fluconazole 87% Echinocandin 13%	IFI 7.4%	AM 80%
Montesinos P. BMT 2015 (63)	retrospective 2001–2013	404	Allo	Voriconazole 65% Itraconazole 25% No/fluconazole 10%	IFI 11%	-

**Abbreviations**: Auto, autologous HSCT; Allo, allogeneic HSCT; AM, attributable mortality; IMI, invasive mold infection; IA, invasive aspergillosis; MSD, matched sibling donor; MUD, matched unrelated donor.

**Table 2:** Studies on antifungal prophylaxis in patients receiving HSCT.

Reference	Study	HSCT	Study drug	Comparator	Main Results
**AZOLES**					
Goodman (13)	Prospective	Autologous allogeneic	**Fluconazole** 400 mg 179 patients	Placebo 177 patients	IFI: 2.8% fluconazole; 15.8% placebo Fewer IFI-related deaths
Slavin (14) Marr (15)	Prospective	Autologous allogeneic	**Fluconazole** 400 mg 152 patients	Placebo 148 patients	IFI: 7%fluconazole; 18% placebo Better OS with fluconazole
Winston (16)	Prospective	allogeneic	**Itraconazole** 400 mg 71 patients	Fluconazole 400 mg 67 patients	IFI: 9% itraconazole; 25% fluconazole Fewer IFI-related deaths (9% vs 18%) More GI side effect with itraconazole
Marr (17)	Prospective	allogeneic	**Itraconazole** 2.5 mg/Kg TID po 151 patients	Fluconazole 400 mg 148 patients	IMI: 5% itraconazole; fluconazole 12% No difference in OS
Wingard (18)	Prospective		**Voriconazole** 200 mg BID 306 patients	Fluconazole 400 mg 295 patients	No difference in IFI rate
Marks (19)	Prospective	allogeneic	**Voriconazole** 200 mg BID 234 patients	Itraconazole 200 mg BID 255 patients	No difference in IFI rate Voriconazole better tolerated
Ullmann (20)	Prospective	Allogeneic with GVHD	**Posaconazole** 200 mg TID 291 patients	Fluconazole 400 mg 288 patients	IFI: posaconazole 2%; fluconazole 8% IA: posaconazole 1%; fluconazole 6%
Wang (22)	Retrospective	Allogeneic	**Posaconazole** 200 mg TID 12 patients	Fluconazole 400 mg 40 patients	IFI: 42% fluconazole; posaconazole 8%
Winston (21)	Retrospective	Allogeneic	**Posaconazole** 200 mg TID 106 patients	-	IFI 7.5%
Sanchet-Ortega (64)	Prospective	Allogeneic	**Posaconazole** 200 mg TID 33 patients	Itraconazole 400 mg 16 patients	IFI: posaconazole 0%; itraconazole 12% FFS: posaconazole 91%; itraconazole 56% OS: posaconazole 91%; itraconazole 63%
Chafteri (23)	Prospective	Allogeneic	**Posaconazole** 200 mg TID 21 patients	ABLC 7.5 mg/Kg once/week 19 patients	IFI: posaconazole 0%: ABLC 5%
**ECHINOCANDINS**					
Van Burik (24)	Prospective	Autologous allogeneic	**Micafungin** 50 mg 425 patients	Fluconazole 400 mg 457 patients	IA: micafungin 1.6%; fluconazole 2.4% No difference in OS
Hiramatsu (65)	Prospective	Autologous Allogeneic	**Micafungin** 150 mg 52 patients	Fluconazole 400 mg 52 patients	No proven-probable-suspected IFI): 94% micafungin; 88% fluconazole
Hashino (66)	Prospective	Allogeneic	**Micafungin** 100 mg 44 patients	Fluconazole 400 mg 29 patients	IFI: 4/41 micafungin; 10/29 fluconazole
Huang (67)	Prospective	Autologous Allogeneic	**Micafungin** 50 mg 136 patients	Itraconazole 2.5 mg/Kg TID po 147 patients	No proven-probable-suspected IFI: 94% micafungin; 88% fluconazole
El-Cheikh (68)	prospective	Allogeneic (Haploidentical)	**Micafungin** 50 mg 26 patients	-	No IFI
Chou (69)	Retrospective	Autologous Allogeneic	**Caspofungin** 35–50 mg 123 patients	-	IFI: 7.3%
**POLYENES**					
Luu Tran (27)	Prospective	Allogeneic with GVHD	**L-AmB** 3 mg/Kg once/week 16 patients	Echinocandins: 12 patients Triazoles: 73 patients	IFI: L-AmB 19%; Echinocandins 17% Triazoles 7%
El-Cheikh (26)	Retrospective	Allogeneic with GVHD	**L-AmB** 7.5 mg/Kg once/week 42 patients	Other antifungal prophylaxis 83 patients	IFI 8% vs 36% IFI-related deaths 0% vs 14%
Koh (70)	Prospective	Autologous Allogeneic	**D-AmB** 0.2 mg/Kg 86 patients	Fluconazole 200 mg 100 patients	IFI: 12% fluconazole; 12.8% D-AmB No difference in OS IFI-related deaths 6% fluconazole 7% D-AmB

**Abbreviations:** L-AmB, Liposomal Amphotericin B; D-AmB, deoxycholate Amphotericin B; ABLC, Amphotericin B lipid complex; IFI, invasive fungal infection; OS, overall survival; GI, gastrointestinal; FFS, fungal-free survival.

**Table 3:** Strength of recommendation on antifungal prophylaxis in allogeneic HSCT recipients

	Pre-Engraftmen Phase					Post-engraftment Phase/GVHD				

	ECIL-5 2013	ESCMID 2014	DGHO 2014	GITMO 2014	NCCN 2015	ECIL-5 2013	ESCMID 2014	DGHO 2014	GITM O 2014	NCCN 2015

**Fluconazole** **400 mg QD**	A I	-	B I	A I	1	A III against	-	-	-	-

**Itraconazole** **400 mg QD**	B I	D I	C I	-	2B	B I	C II	C I	-	-

**Posaconazole**										
**Oral sol 200 mg q8h**	B II	B II	B II	-	2B	A I	A I	A I	A I	1
**Tab 300 mg QD**	B II	-	B II	-	2B	A I	-	A II	-	1

**Voriconazole** **200 mg q12h**	B I	C I	B I	B I	2B	B I	C II	C II	B I	2B

**Caspofungin** **50 mg QD**	No data	-	-	-	-	-	-	-	C III	2B

**Micafungin** **50 mg QD**	B I LR-mold C I HR-mold	C II	B I	B I	1	C II	C III	C II	C III	2B

**L-AmB**	C II	-	-	C III	2B	C II	-	-	C III	-

**Areosol AmB**	C III	-	-	-	-	-	-	-	C III	-

**ECIL-5**: www.kobe.fr/ECIL5antifungalprophylaxis

**ESCMID:** www.escmid.org

**DGHO:** Tacke D, Buchheidt D, Karthaus M. et al. Primary prophylaxis of invasive fungal infections in patients with haematologic malignancies. 2014 update of the recommendations of the Infectious DiseasesWorking Party of the German Society for Haematology and Oncology. Ann Hematol 2014, 93:1449–1456

**GITMO:** Ref 7

**NCCN:** www.NCCN.org

**Table 4:** Clinical trials comparing antifungal agents as empiric antifungal therapy in patients with febrile neutropenia

		Author/year	Study drugs	No. patients	IFI	Success rates
**d-AmB vs no Therapy**						
		Pizzo, 1982 (34)	d-AmB	18	6%	
			antibiotics	16	31%	ND
			No Tx	16	0%	
		EORTC, 1989 (35)	d-AmB	68	1%	69%
			antibiotics	64	9%	53%

**Azoles**						
	Fluco vs d-AmB					
		Viscoli, 1996 (71)	Fluco	56	ND	75%
			d-AmB	56		66%
		Malik, 1998 (36)	Fluco	52	ND	56%
			d-AmB	48		46%
		Winston, 2000 (37)	Fluco	107	8%	68%
			d-AmB	106	6%	67%
	Itra vs d-AmB					
		Boogaerts, 2001 (38)	Itra	192	3%	47%
			d-AmB	192	3%	38%
	Vorico vs L-AmB	Walsh, 2002 (39)	Vorico	415	1.9%	26%
			L-AmB	422	5%	31%

**AmB lipid formulations**						
	L-AmB vs d-AmB					
		Walsh, 1999 (40)	L-AmB	343	3%	50%
			d-AmB	344	8%	49%
		Prentice, 1997 (41)	d-AmB 1 mg/Kg	102	2%	49%
			L-AmB 1 mg/Kg	118	2.6%	58%
			L-AmB 3 mg/Kg	118	1.7%	64%
	ABLC vs L-AmB	Wingard, 2000 (72)	ABLC 5 mg/Kg/d	78	3.8%	33%
			L-AmB 3 mg/Kg/d	85	3.6%	40%
			L-AmB 5 mg/Kg/d	81	2.5%	42%

**Echinocandins**						
	Caspofungin vs L-AmB					
		Walsh, 2004 (42)	Caspofungin	556	5%	34%
			L-AmB	539	4%	34%

**Abbreviations:** AmB, amphotericin-B; d-AmB, amphotericin-B deoxycholate; ND, not documented; L-AmB, liposomal AmB; ABLC, amphotericin B lipid complex; fluco, fluconazole; itra, itraconazole; vorico, voriconazole.

**Table 5:** Summary of the studies analyzing first-line antifungal therapy in hematologic patients.

	Herbrecht NEJM 2002 (50)		AMBILOAD	EORTC		COMBO		SECURE	
			Cornely CID 2007 (73)	Herbrecht BMT 2010 (74)	Viscoli JAC 2009 (75)	Marr Ann Int Med 2015 (51)		Maertens Lancet 2015 (52)	

	Vori	D-AmB	L-AmB 3 mg/Kg	Caspo		Vori+Anidula	Vori	Isavuc	Vori

**No. patients**	144	133	107	24	61	135	142	123	108

**Median age**	48	50	51	50	64	52	52	51	51

**Acute leukemia**	40%	45%	-	-	63%	44%	45%	50%	58%

**Allogeneic HSCT**	26%	23%	16%	100%	0	33%	30%	21%	20%

**Favourable response**	53%	32%	50%	42%	33%	33%	43%	35%	36%

**Servival at 12 weeks**	71%	58%	72%	50%	53%	71%	61%	72%	64%

**Abbreviations:** Vori, voriconazole; D-AmB, amphotericin B deoxycholate; L-AmB, liposomal amphotericin B; Caspo, caspofungin; Anidula, anidulafungin; Isavuc, isavuconazole.
